# Reflections on Participation in a Trial on Hydroxychloroquine as Prevention for COVID-19 among Health Workers in Niger

**DOI:** 10.4269/ajtmh.22-0606

**Published:** 2023-08-14

**Authors:** Youssouf Kabore, Renaud Vatrinet, Ousmane Guindo, Souleymane H. Moussa, William H. K. Schilling, Rebecca F. Grais

**Affiliations:** ^1^ Epicentre Niger, Niamey, Niger;; ^2^ Research Department, Epicentre Paris, Paris, France;; ^3^ Service de Pneumologie, Hôpital National Amirou Boubacar Diallo (Lamorde), Niamey, Niger;; ^4^ Centre for Tropical Medicine & Global Health, Nuffield Department of Medicine, University of Oxford, Oxford, United Kingdom;; ^5^ Mahidol Oxford Tropical Medicine Research Unit, Faculty of Tropical Medicine, Mahidol University, Bangkok, Thailand

## Abstract

In 2020, severe acute respiratory syndrome coronavirus 2 (SARS-CoV-2), a rapidly emerging virus causing the coronavirus disease 2019 (COVID-19) pandemic, had no known effective prophylaxis and no widely available proven effective antiviral treatment. Hydroxychloroquine/Chloroquine was identified as an early potential therapeutic candidate drawing on evidence from reports of both in vitro and in vivo testing. A multicountry placebo-controlled randomized trial was set to evaluate the use of hydroxychloroquine/chloroquine to prevent infection in healthcare workers and staff working in a health facility involved in COVID-19 management. One of the sites of this trial was in Niger. In Niger, of the 240 persons who were provided information about the study and with whom participation was discussed, only five participants provided their informed consent. In this article, we describe the key difficulties encountered in the conduct of this trial from the perspective of the site study team. Among the difficulties, we recognize that the epidemic context, controversy surrounding hydroxychloroquine, vaccine rollout, participants’ perspectives, and trial design had a major impact on participation.

## INTRODUCTION

In 2020, there was no proven effective prophylaxis and no globally available proven effective antiviral treatment of coronavirus disease 2019 (COVID-19). Vaccines were not yet available. In vitro studies suggested that hydroxychloroquine (HCQ) was effective in inhibiting severe acute respiratory syndrome coronavirus 2 (SARS-CoV-2) in a cell culture model.^1^ However, the two largest drug trials assessing the potential of HCQ in treatment, the UK RECOVERY and the WHO SOLIDARITY trials, failed to detect any benefit among hospitalized patients.^2^^,^^3^ On the other hand, there remained substantial uncertainty, and thus clinical equipoise, regarding the efficacy of HCQ in preventing COVID-19, pointing to the need for larger, global clinical trials to evaluate its prophylactic properties.^4^

In the setting of this uncertainty, a global multicentric, double-blind, randomized, placebo-controlled trial (COPCOV) was set to evaluate the use of HCQ to prevent healthcare workers and staff working in a health facility involved in COVID-19 management from being infected and developing symptomatic disease. After informed consent was obtained, participants were randomly assigned to receive either 200 mg HCQ daily or placebo for 90 days. The primary outcome was the incidence of symptomatic COVID-19 infection. In Niger, potential participants were recruited from Lamorde Hospital, in the capital Niamey.

Community engagement activities started in mid-January 2021 and included meetings with administrative and health authorities. The first step in the process was to discuss the study with all heads of departments in the hospital and ensure that their questions and any concerns were addressed. Study information was extended to all hospital staff on February 10, 2021. In addition to these meetings, study staff were permanently present at the hospital to answer questions and discuss participation in a confidential manner.

Of the 240 persons with whom participation was discussed, only five participants provided their informed consent through mid-April 2021. Here, we describe the key difficulties encountered in the conduct of this trial from the perspective of the site study team, with four major considerations: the context, the investigational product, the participants, and the design of the trial itself.

## THE CONTEXT

**Figure 1.**
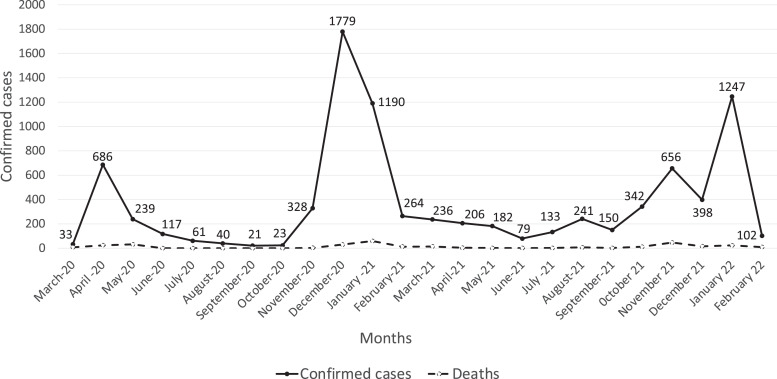
Covid-19 confirmed cases and deaths in Niger from March 2020 to February 2022.

The incidence and mortality reports from COVID-19 in Africa have varied widely from country to country. In Niger, confirmed cases were low.^5^ Three waves of COVID-19 cases in April 2020, December 2020, and January 2022 (Figure 1) were detected. Between March 2020 and February 2022, Niger reported a total of 8,756 cases and 307 deaths. Most cases were reported among migrants and travelers requiring a polymerase chain reaction (PCR) test before or after traveling. Although official data could have been underestimated, the perceived risk of COVID-19 was not considered a priority by the public. This was further illustrated, and certainly further nourished, by media releases that described Niger as the country that COVID-19 had forgotten.^6^

Niger received a first shipment of 400,000 doses of COVID-19 vaccines produced by Sinopharm (BBIBP-CorV) on March 21, 2021^7^ and 355,000 doses of AstraZeneca (AZD1222) vaccine procured through the COVAX initiative.^8^ Vaccines were initially prioritized for healthcare workers and high-risk persons and later extended to all adults.

As vaccines became available to healthcare workers, their priority shifted from other potential prophylaxis options toward vaccines. On April 20, 2021, the COPCOV trial site was put on pause, and the protocol was amended to account for COVID-19 vaccination, which was then being given priority. Anyone who received a COVID-19 vaccination would not be eligible for enrollment; however, participants who decided to receive a vaccine after enrollment would hitherto be followed through D90 (90 days after) or 28 days after the second dose of vaccine, whichever came later; they would also have venous blood drawn 28 days after both the first and second vaccine doses and would no longer take study medication.

Despite an attempt to adapt to the availability of vaccine, enrollment did not change, highlighting additional reasons for low participation.

## THE INVESTIGATIONAL PRODUCT: HCQ

Hydroxychloroquine is an inexpensive and globally accessible drug indicated for the treatment of rheumatic diseases and as both a treatment and a prophylaxis for malaria.^9^ Given the known mechanism of action of HCQ and its widespread availability and history of past use, there was scientific interest in exploring its potential use as a therapeutic or prophylactic.^10^ Observational studies conducted in France and other countries lent further hope to the potential benefits of HCQ. Although no reliable trials had yet been conducted at the outset, HCQ received significant media attention, and eventually, emergency use authorizations (EUAs) were issued in many countries,^11^ together with a remarkable rise in both the off-label use of HCQ and interest within the clinical research community.^12^ As for many African countries, HCQ was in the national protocol in Niger as treatment against COVID-19.

Despite the attention and hope generated by HCQ in other parts of the country and the subregion, at the study site, information became widely available that HCQ was not a viable option for treatment. Notably, the U.S. Food and Drug Administration’s withdrawal of the EUA was widely shared by the media and on social media locally.

## THE PARTICIPANTS

Although a formal study of reasons for nonparticipation was not conducted, discussions revealed that potential participants did not feel at risk of contracting COVID-19. Yet, the later acceptance of vaccination was much more pronounced than the acceptance of participating in the trial with HCQ, suggesting that nonparticipation may have been motivated more by unwillingness to use the experimental product—whether because of controversy around its use or the necessity for strict adherence to the protocol requiring daily intake during 90 days—than by other factors.

In addition, the poor enrollment can also be explained by the participants’ desire to receive what was felt to be appropriate compensation for their participation in the research. This is called *benefit sharing*, and it includes information and knowledge collected through the research process, as well as the social benefits identified in the study.^13^ However, the social benefit depends on the context. Over the past decade, researchers have shown increased interest in conducting clinical trials and other health-related research in resource-poor settings.^14^ For people in these areas, participating in such research is often beneficial because it can give them an otherwise rare opportunity to access medical care.^15^ In our context, participants were healthcare workers who may therefore have greater access to care services and knowledge sources than nonmedical persons. The inability to get approval to enroll non-healthcare workers—a larger pool of potential participants whose attitudes and access to vaccines may have differed—into the study may have affected overall recruitment, as other sites that had struggled with enrollment (e.g., Pakistan and Nepal) were able to recruit more participants once non-healthcare workers became eligible. Potential participants may also have reached an informal consensus on nonparticipation in this study, possibly based on negative local opinion concerning the experimental product or the research protocol, generating an unwillingness to go against prevailing opinions.

## THE DESIGN

Conducting clinical research during epidemics is challenging because epidemic dynamics change, public health interventions adapt rapidly, and the scramble for preventive and curative solutions continually generates promising new interventions while discarding others. This poses major methodological challenges, as the various changes must be systematically taken into account to continue ensuring both the scientific rigor and the usefulness of the study results.

Adaptive platform trials have been put forward as a potential alternative to a priori designs. Both the RECOVERY and SOLIDARITY trials were platform trials. One of the main advantages is that it is possible to discontinue evaluation of suboptimal arms (investigational product[s]) and redirect resources to the most promising ones, with an efficient use of a single “common” control arm, hence contributing not only to effective knowledge generation but also to trial efficiency.^16^ However, although such designs may be appropriate and efficient for certain treatment trials for COVID-19, prophylaxis has its own specificities. First, platform trials are often open-label. Open-label studies may generate greater willingness to participate than blinded trials, as participants know the treatment they are assigned, although non-negligible bias may be introduced. In COPCOV, this was more important, as the endpoint examined was the presence of symptoms (triggering PCR confirmation of COVID-19), which is more subjective than other endpoints such as hospitalization and death. This argues for placebo controls, which can take considerable time to produce. Second, in platform trials, close to real-time information on endpoints is needed to ensure that arms are added and discarded promptly. Real-time diagnostic tests for SARS-CoV-2 were not widely available at that time, and there was concern that using scarce diagnostics for the study might put additional strain on already overburdened health systems. Third, trial arms may quickly become noncomparable with time, especially when the exposure risk and infection rate change over the course of the trial, such as during the COVID pandemic.^16^^–^^18^ Hence, the different trial arms need to be randomized concurrently throughout the study. Non-concurrent groups, including the control group, may deeply bias the result of the trial.^17^ To conclude, the barriers and risks of platform trials made this methodological approach inappropriate for our study.

## CONCLUSION

There are many aspects that may explain low participation in the COPCOV study in Niger. First, Niger had a very low number of confirmed cases and deaths due to COVID-19, so it was not perceived as a priority problem. Second, the availability of vaccines with proven efficacy and no need for repeated daily doses may have been perceived as being in direct competition with the use of the investigational product. Third, controversies about HCQ were widely publicized, leading to the development of negative opinions by participants about its effectiveness. Fourth, because participants were healthcare professionals, they belonged to a medical community that may have been more likely to develop specific opinions and beliefs around a particular health topic, which may even have been at odds with the investigator’s perspective, and to resist the study team’s communication efforts. Many of these issues affected all sites in the study, which were able to recruit 4,652 participants in 25 sites in 11 countries, the majority of them in Africa (including Benin, Côte d’Ivoire, and Mali), although this total was substantially less than the planned number. This confirms that the conduct of clinical research to find a curative or preventive solution to an ongoing global pandemic was subject to context-specific issues, such as the emergence of alternative products, parallel and competing public health interventions, and public perceptions, whose complex interplay may have led to the study’s success or failure in a particular site. Although adaptive platform trials may hold promise for treatment, the challenges for prophylaxis are numerous.
